# HBV/HIV co-infection and APOBEC3G polymorphisms in a population from Burkina Faso

**DOI:** 10.1186/s12879-016-1672-2

**Published:** 2016-07-22

**Authors:** Tegwinde Rebeca Compaore, Birama Diarra, Maleki Assih, Dorcas Obiri-Yeboah, Serge Theophile Soubeiga, Abdoul Karim Ouattara, Damehan Tchelougou, Cyrille Bisseye, Didier Romuald Bakouan, Issaka Pierre Compaore, Augustine Dembele, Wendkuuni Florencia Djigma, Jacques Simpore

**Affiliations:** Pietro Annigoni Biomolecular Research Centre (CERBA)/LABIOGENE, University of Ouagadougou, Ouagadougou, Burkina Faso; Department of Microbiology and Immunology, School of Medical Sciences, University of Cape Coast, Cape Coast, Ghana; Permanent Secretary against Aids and sexually transmitted diseases, Ouagadougou, Burkina Faso

**Keywords:** HIV-1/HBV co-infection, APOBEC3G variants, Real time PCR, Burkina Faso

## Abstract

**Background:**

Apolipoprotein B mRNA editing enzyme catalytic polypeptide-like 3G (APOBEC3G) is a potent host defense factor, which interferes with HIV-1 and HBV. Our study had three objectives, to screen a population of HIV-1 infected and uninfected patients in Burkina Faso for HBV, to screen the population for APOBEC3G variants rs6001417, rs8177832, and rs35228531 previously described, and to analyze the effect of these three variants and their haplotypes on HIV-1/HBV co-infection in Burkina Faso.

**Methods:**

HBV detection was performed on samples from HIV-1 infected and uninfected subjects using rapid detection tests and real-time PCR. APOBEC3 genotyping was done by the TaqMan allelic discrimination method. Fisher Exact test, Odds ratio (OR), confidence intervals (CI) at 95 %, Linkage disequilibrium (LD) summary statistics and haplotype frequencies were calculated.

**Results:**

The prevalence of HBV was 56.7 % among HIV-1 positive patients of our study while it was about 12.8 % among HIV-1 seronegative subjects. Genotype E was the genotype of HBV present in our hepatitis B positive samples. Minor allele frequencies of rs6001417, rs8177832, and rs35228531 were higher in seronegative subjects. The T minor allele of variant rs35228531 was protective against HIV-1/HBV co-infection with OR = 0.61, 95 % CI (0.42–0.90), *p* = 0.013. There was also an association between the GGT haplotype and protection against HIV-1/HBV co-infection, OR = 0.57, 95 % CI (0.33–0.99), *p* = 0.050. The other haplotypes present in the population were not statistically significant. There minor allele T of the rs35228531 was protective against HIV mono-infection OR = 0.53, 95 % CI (0.3–0.93), *P* = 0.030. But there was no effect of protection against HBV mono-infection.

**Conclusion:**

APOBEC3G through its variants rs6001417, rs8177832, and rs35228531, in this study interferes with HIV-1/HBV co-infection could be due the HIV-1 mono-infection in a population from Burkina Faso.

## Background

According to the World Health Organization (WHO), a significantly high number of the adult population in sub-Saharan Africa are infected with Hepatitis B virus (HBV) [1]. In Burkina Faso, the prevalence of HBV in the adult population is about 14.4 % among the general population [2]. At the end of 2013 about 35 million people were living with Human Immunodeficiency Virus (HIV-1) worldwide, with sub-Saharan Africa having the highest toll of this infection as it harbors 71 % of the infected persons worldwide [3]. Globally, about 6-14 % person infected by HIV-1 have the hepatitis B surface antigen (HBsAg) [4–6]. Worldwide, 3 to 6 million of individuals infected with HIV-1 also have chronic hepatitis B [7]. In Sub-Saharan Africa HIV-1/HBV frequency varies from 0 % to >28.4 % [8, 9]. HIV-1 infection has been associated with a high prevalence of HBV infection in Burkina Faso [10, 11]. Co-infection of HIV-1 and HBV is due to the common routes of transmission and increases the morbidity and mortality of the two infections. People having HIV-1/HBV co-infection have an increased complexity of treatment and this contributes to poor medical outcome [12]. Efforts have been focused on the host genetic factors (genetic mutations) that affect disease progression in HIV/AIDS [13] and hepatitis B chronicity [14] such as APOBEC3G. In fact, Apolipoprotein B mRNA editing enzyme catalytic polypeptide-like 3G (APOBEC3G) is a potent host defense factor, which interferes with HIV-1 [15, 16] and HBV [17, 18]. The APOBEC3G protein is incorporated into newly synthesized viral particles, in the absence of the virion infectivity factor (vif), and hypermutates viral DNA by deamination which transforms cytosine (C) to uracil (U). APOBEC3G enzymes restricts hepatitis B via the nascent minus DNA strand deamination [14, 19]. APOBEC3G polymorphisms, such as rs8177832 (H186R), are thought to be associated with HIV-1 subtype B and C pathogenesis in some ethnic groups [20, 21], though this association is not seen in other populations [22, 23]. In our previous study we have found that the minor alleles of APOBEC3G variants), rs6001417, rs8177832 (H186R), and rs35228531 were associated with protection against HIV-1 infection [24]. Here we investigated the prevalence of HBV in an HIV-1 infected subjects and the association of HIV-1/HBV co-infection with APOBEC3G variants in Burkina Faso.

## Methods

### Study population

Four hundred and twenty-four (424) people (150 HIV-1 positive and 274 HIV-1 negative) were tested for the carriage of hepatitis B surface antigen (HBsAg) and hepatitis C. The HIV-1 seronegative individuals were not vaccinated against HBV.

Eighty-Five (85) HIV and HBsAg positive (co-infected) and 239 individuals who are seronegative for both HIV-1 and HBV were further analyzed. All the subjects were recruited from the Pietro Annigoni Biomolecular Research Center (CERBA).

### Sample collection, HIV-1, HBsAg detection and HIV-1 and HBV Plasma Viral load quantification

Plasma samples obtained by venipuncture were tested for HBsAg carriage using the ABON test and for HIV-1 and HBV co-infection, the HCV/HBV/HIV Real Time PCR kit was used (Sacace Biotechnologies, Como, Italy). CD4 cells were enumerated using the BD FACSCount CD4 Reagent kit on a BD FACS COUNT (Becton Dickinson, San Jose).

HIV-1 viral load was determined using the Abbott HIV-1 Real Time Quantitative kit (Promega, USA) on the Abbott *m*2000rt (Abbot Laboratories, Illinois) according to the manufacturer’s protocol. HBV viral load was determined using Genesig HBV Real Time Quantitative kit (Primerdesign, Southampton, United Kingdom).

### DNA extraction and APOBEC3G genotyping

For HIV-1 and HBV detection, viral RNA/DNA was extracted from plasma using the Ribo-Sorb kit (Sacace Biotechnologies, Como, Italy) according to the manufacturer instructions. Genomic DNA was extracted from leucocytes using the “DNA Rapid Salting-Out” technique as described by Miller et al. [25]. SNP selection was based on the most relevant APOBEC3G variants, rs8177832 (H186R), rs35228531 and rs6001417 described elsewhere [21]. The three markers of APOBEC3G studied were genotyped using standard TaqMan SNP assays (ABI, Foster City, CA) run on the 7500 Fast Real-Time PCR Systems (Life Technologies, California, USA).

### HBV genotyping

For HBV genotyping purposes, the HBV DNA was amplified by multiplex PCR by using two different master mixes with three sets of primers of the genotypes ABC and DEF [26] were used. These mixes contained 25 μl of Taq polymerase, MgCl2, dNTPs, buffer 1X, reverse, forward primers, 0.4 μl of water nuclease free and 5 μl of extracted DNA. The latter was amplified using the Gene Amp PCR System 9700 (Applied Biosystems, USA) with the following cycling conditions: 95 °C during 15 mn followed by 35 cycles of 94 °C during 1mn, 58 °C during 1mn and 72 °C during 1mn; finally with 72 °C during 10 mn. PCR products were run through a 2 % agarose gel and revealed under UV.

### Ethical considerations

Approval for the study was obtained from the National Health Ethic Committee of Burkina Faso (reference number 2014-7-086 of July, 7^th^ 2014). All study participants or guardians gave their free written and informed consent according to the Helsinki Declarations.

### Statistical analysis

SPSS version 20.0 was used for data analysis. Power-Marker software version 3.25 was used for the determination of the Hardy-Weinberg equilibrium and the calculation of allele and genotype frequencies. Changes were considered statistically significant at *p* ≤ 0.05, using the Fisher Exact test. Odds ratio (OR) and confidence intervals (CI) at 95 % were calculated to estimate the associations of HIV-1/HBV co-infection with the rs6001417, H186R, and rs35228531 polymorphisms using Epi Info 7. Logistic regression was performed on all SNPs with statistically significant allele or genotype tests, associating additive, dominant and recessive models with minor allele as the risk allele.

Linkage disequilibrium (LD) was characterized and haplotype frequencies were computed using Power-marker [27], Haploview [28], and SNPStats [29] statistical softwares. Descriptive summary statistics were calculated using Haploview [28]. Power Marker, Haploview and SNPStats use the Expectation Maximization (EM) algorithm to determine haplotype frequency distributions in cases of unknown phase. Only haplotypes with a minimum frequency of 10 % were considered in the analysis.

## Results

### Clinical and biological features

The prevalence of HBV was 56.7 % in the HIV-1 positive patients of our study while it was 12.8 % among the HIV-1 seronegative subjects.

All study subjects, tested negative for HCV.

Gender and age distribution are presented in Table 1. There was no significant difference between the age and gender among the two groups (HIV-1 positive and negative). The viral genotypes of hepatitis B present among the study group was genotype E (100 %) (Table 1).

**Table 1:** Study participants’ baselines data

	HIV(+)/HBV(+) *n* = 85	HIV(+) (*n* = 35)	HBV(+) (*n* = 65)	HIV(−)/HBV(−) *n* = 239
Detectable HBV Viral Load	35	–	20	–
Mean HIV-1 Viral Loads	65,963.68 +/−20,2480	6,758.13+/− 30368.61	–	–
Mean CD4 counts	423.45 +/−300.33	375.12 +/− 277,20	N/A	N/A
HBV genotypes E n (%)	100	–	100	–

+/− *SD* standard deviation values, *N/A* not available

### Alleles and Genotypes frequencies

APOBEC3G variants rs6001417, rs8177832 (H186R) and rs35522832 genotypes are shown in Table 2. The 3 SNPs were in Hardy-Weinberg equilibrium in cases and controls (Table 2).

**Table 2:** Frequencies of APOBEC3G gene polymorphisms (cases vs. controls)

					HIV (+) versus HIV(−)/HBV(−)			HBV(+) versus HIV(−)/HBV(−)			HIV(+)/HBV(+) versus HIV(−)/HBV(−)		
Polymorphisms	HIV(+)	HBV(+)	HIV(+)/HBV(+)	HIV(−)/HBV(−)	OR	95 % CI	*p*-Value	OR	95 % CI	*p*-Value	OR	95 % CI	*p*-Value
	(*n* = 35)	(*n* = 65)	(*n* = 85)	(*n* = 239)									
rs6001417													
CC, n (%)	10 (28.57)	15 (0.23)	26 (30.59)	64 (0.27)	1.09	0.5–2.4	0.84	0.82	0.43–1.56	0.63	1.2	0.7–2.07	0.57
CG, n (%)	17 (48.57)	40 (0.61)	48 (56.47)	114 (0.48)	1.03	0.51–2.1	1	1.75	1.01–3.07	**0.05**	1.47	0.89–2.42	0.13
GG, n (%)	8 (22.86)	10 (0.15)	11 (12.94)	61 (0.25)	0.415	0.37–2	0.84	0.415	0.37–2	0.84	0.415	0.207–0.83	**0.02**
G vs C, n (%)	33 (0.94)	60 (.92)	35 (0.41)	236 (0.99)	0.9	0.55–1.51	0.8	0.82	0.55–1.2	0.32	0.759	0.5–1.06	0.06
HWE	0.74	0.16	0.20	0.38	–	–	–	–	–	–	–	–	–
rs8177832													
AA. n (%)	10 (28.57)	15 (0.23)	27 (31.76)	69 (28.87)	0.98	0.45–2.16	1	0.74	0.39–1.4	0.43	1.15	0.67–1.96	0.7
AG. n (%)	18 (0.51)	40 (0.61)	45 (52.94)	111 (0.46)	1.22	0.60–2.48	0.59	1.84	1.05–3.23	**0.04**	1.34	0.82–2.2	0.26
GG. n (%)	7(0.2)	10 (0.15)	13 (15.30)	59 (0.25)	0.76	0.32–1.84	0.67	0.55	0.26–1.15	0.13	0.53	0.28–1.02	0.07
G vs A. n (%)	32 (0.91)	60 (0.92)	36 (42.35)	229 (0.96)	0.9	0.55–1.51	0.8	0.82	0.55–1.2	0.32	0.77	0.54–1.09	0.15
HWE	0.98	0.16	0.59	0.21	–	–	–	–	–	–	–	–	–
rs35228531													
CC. n (%)	18 (0.51)	18 (0.51)	40 (47.05)	84 (35.15)	1.95	0.96–4	0.09	1.15	0.65–2.03	0.66	1.64	0.99–2.71	0.07
CT. n (%)	15 (0.43)	15 (0.43)	38 (44.70)	113 (0.47)	0.84	0.4–1.71	0.17	0.95	0.55–1.65	0.89	0.93	0.57–1.53	0.80
TT. n (%)	2 (0.06)	2 (0.06)	7 (8.25)	42 (0.17)	0.28	0.06–1.23	0.09	0.85	0.40–1.80	0.85	0.4	0.17–0.9	**0.04**
T vs C. n (%)	19 (0.54)	19 (0.54)	25 (29.88)	197 (0.82)	0.53	0.3–0.93	**0.03**	0.89	0.6–1.33	0.615	0.61	0.42–0.90	**0.01**
HWE	0.88	0.98	0.86	0.55	–	–	–	–	–	–	–	–	–

*HWE* Hardy-Weinberg equilibrium, *OR* odds ratio, *CI* confidence interval. Significant *p*-Values are in bold

The minor genotype GG of rs6001417 (Table 2) was more frequent among HIV-1(−)/HBV (−) compared to HIV-1(+)/HBV (+) subjects, and the difference was statistically significant: OR = 0.42, 95 % CI (0.21–0.83), *p* = 0.017. The results show that GG genotype seems to be protective. The G allele of rs6001417 was also more frequent among HIV(−)/HBV(−) than HIV(+)/HBV(+) but the difference was not statistically significant: OR = 0.759, 95 % CI (0.5–1.06), *p* = 0.06.

We had the GG genotype of the rs8177832 which has a low frequency in controls compared to cases but the difference was not significant: OR = 0.53, 95 % CI (0.28–1.02), *p* = 0.07 (Table 2). The minor allele G had the same profile as the GG genotype for cases and controls but the difference was not statistically significant, OR = 0.77, 95 % CI (0.54–1.09), *p* = 0.15.The normal CC genotype of the rs35522832 was more frequent among cases than controls, this difference was not statistically significant: OR = 1.64, 95 % CI (0.99–2. 71), *p* = 0.07. The normal C allele was also more frequent in cases than in controls, this variation was statistically significant with OR: 1.61, 95 % CI (1.11–2.35), *p* = 0.013 and seem to almost double the risk of infection. While the TT genotype had a higher frequency in controls compared to cases, the difference was statistically significant: OR: 0.4, 95 % CI (0.2–0.9), *p* = 0.04, and demonstrated a protective effect. The T allele had the same protective profile: OR = 0.61, 95 % CI (0, 42–0. 90), *p* = 0.013.

The genotypes and alleles of different variants of APOBEC3G were compared between HIV mono-infected patients and healthy controls are mostly significant for the minor allele T of the rs35228531 (Table 2).

When comparing healthy controls to HBV mono-infected patients’ genotypes and alleles, there was only an association with risk of infection with the rs6001417 heterozygous CG and the AG of rs8177832 (Table 2).

The association between the 3 single loci and HIV-1/HBV co-infection status based on the additive, dominant and recessive model seem to show that normal genotypes of are associated based on an additive model, with an increased risk of co-infection especially for rs6001417 OR = 2.4 95 % CI (1.21–4.7), *p* = 0.01 and rs35228531 OR = 2.66, 95 % CI (1.16–6.07), *p* = 0.02 (Table 3).

**Table 3:** Association between 3 single loci and HIV-1/HBV co-infection status. Based on additive, dominant and recessive models

Markers	Models	OR	95 % CI	*p*-Value
rs6001417	C/C (referent). C/G. G/G. additive	2.4	1.21–4.7	**0.01**
	C/C vs. C/G & G/G. dominant	1.20	0.7–2.07	0.5
	G/G vs. G/G&G/G. recessive	0.4	0.2–0.83	**0.01**
rs8177832	A/A (referent). A/G. G/G additive	1.88	0.99–3.57	0.06
	A/A vs. A/G &G//G. dominant	1.15	0.67–1.96	0.62
	G/G vs. A/A &A/G. recessive	0.53	0.27–1.017	0.07
rs35228531	C/C (referent). C/T. T/T. additive	2.66	1.16–6.07	**0.02**
	C/C vs. C/T & T/T. dominant	1.64	0.99–2.71	0.07
	T/T vs. C/C & C/T. recessive	0.4	0.17–0.92	**0.04**

*OR* odds ratio, *CI* confidence interval. Significant *p*-Values are in bold

### Haplotypes in cases and controls

Haploview software analysis also detected six (6) haplotypes and their frequencies are shown in Table 4. The frequency of the GGT haplotype is higher among controls (39.27 %) than among cases (27.38 %), this difference is statistically significant: OR = 0.57, 95 % CI (0.33–0.99), *p* = 0.05. The other haplotypes did not have significant differences.

**Table 4:** Haplotypes of HIV/HBV and controls

Haplotype	HIV(+)/HBV(+)	HIV(−)/HBV(−)	OR	CI	*p*-Value
CAC	0.54	0.51	1.27	0.77–2.1	0.38
GGC	0.11	0.11	1.45	0.62–3.73	0.37
GGT	0.27	0.31	0.57	0.33–0.99	**0.05**
CGC	0.04	0.02	2.15	0.47–9.81	0.40
GAT	0.02	0.02	1.41	0.25–7.87	0.65
GAC	0.01	0.01	0.94	0.1–9.1	1

*OR* odds ratio, *CI* confidence interval. Significant *p*-Values are in bold

HIV-1 mono-infected patients had haplotypes with stronger linkage disequilibrium compared to HBV mono-infected patients.

## Discussion

We investigated the distribution of three genetic variations within the APOBEC3G gene in HIV-1, HBV positive, HIV-1/HBV co-infected and healthy control individuals from Burkina Faso. The genotype of HBV present in our study was genotype E for all the HBsAg positive samples. This finding confirms the predominance of HBV genotype E in West Africa [30]. The prevalence of HBV infection of 12.8 % in HIV seronegative subjects is similar to the prevalence found by Tao et al. [2]. HIV-1 positive patients had a higher rate of HBV infection (56.7 %) compared to HIV negative subjects and is similar to the HIV-1/HBV co-infection data from Sub-Saharan Africa [31]. Furthermore, the genotype E of HBV is frequent in HIV/HBV co-infection as mentioned by Yousif et al. [32]. Although HIV/AIDS and HBV affects Sub-Saharan Africa at a high rate, no study has been conducted on the definitions of AIDS and HBV co-infection restriction genes on native populations, in which genotype frequencies depend on ethnic background.

HCV infection was not found in our study group, although HCV co-infection is very common in HIV-1-infected intravenous drug users especially in most parts of Europe and USA, but not so much in Sub-Saharan Africa [33].

In this study, we describe the frequency of 3 SNP genotypes and their haplotypes on HIV-1, HBV and especially on HIV-1/HBV co-infected subjects compared to controls. There have been very few studies on rs6001417 and rs35228531 since their identification by Reddy et al. 2010 [21].

The H186R mutation of APOBEC3G has been reported in the literature to be associated with an accelerating effect on disease progression in African Americans infected with HIV-1 at a frequency of 37 % [20]. In our precedent study we have found that the minor alleles of APOBEC3G variants rs6001417, rs8177832 (H186R), rs35228531 were associated with protection against HIV-1 infection. The latter variants frequencies were respectively 44.3, 43.8 and 32.8 % in HIV-1 infected patients from Burkina Faso [24] and also in a South African cohort infected with HIV-1 with a frequency of 30.7 % [21]. The H186R was studied in a Morrocan HBV chronically infected patients, found a low frequency of G and a higher frequency of A but there was no significant difference allele frequencies between cases and controls [34]. In our study, this mutation occurred at a frequency of 48.12 % in healthy controls compared to 42.35 % in co-infected HIV-1(+)/HBV (+) patients.

The minor allele T allele of the rs35228531 had a protective profile so were the remaining two alleles G, G of rs6001417 and rs8177832 (Table 2). The latter minor alleles had an odd ratio lower than one but this was not statistically significant. The risk of co-infection with HIV-1/HBV was also reduced for the carriers of genotypes GG and TT of rs6001417 and rs35228531 (Table 2). Furthermore, there was almost a double risk of HIV-1/HBV co-infection for the carriers of the C allele of rs35228531 OR: 1.61, 95 % CI (1.11–2.35), *p* = 0.013.

The minor allele T of patients mono-infected with HIV had a protection effect when compared with the healthy controls, but there was no significant effect with the genotype TT. For the patients mono-infected with HBV, rs6001417 and H186R had a risk of infection increased with the heterozygous carriers CG and AG respectively, but no effect for the minor genotypes. Both heterozygous genotypes may favor HBV infection which in turn is a risk factor for the development of hepatocellular carcinoma (HCC). Indeed, a previous study had suggested that the expression of APOBEC3G is a risk factor for HCC development and survival [35]. Furthermore, heterozygote genotype of a gene could influence a susceptibility to an infection, such as that of TAP1 which may decrease a susceptibility to human papilloma virus (HPV) infection but can increases susceptibility to the development of esophageal cancer among the Kazakh populations [36].

We compared the APOBEC3G alleles between mono- and co-infection and found no significant difference for the polymorphisms distribution. These results can be attributed in part to the clinical status of individuals infected with HBV. Almost all the subjects of this study were asymptomatic carriers of HBV. A previous study has suggested that polymorphisms of APOBEC3G do not predispose to chronicity but could influence the persistence of HBV infection [34].

We observed evidence for haplotype-specific associations in the co-infected group compared to control subjects. In fact for the GGT haplotype, there was a protective effect against HIV-1/HBV co-infection (Table 4). The high linkage disequilibrium existing between the alleles imply that the GGT haplotype seems to have a protective effect against HIV-1/HBV co-infection.

We have found haplotypes associated with HIV and HBV mono-infections but when compared to healthy controls, there was no significant association. The sample size of our study population could explain these results.

Furthermore there were no significant association between haplotypes and the increased risk of being infected as it was found by Compaore et al. [24] in a HIV-1 positive population.

## Conclusion

This study suggests that APOBEC3G is a susceptibility gene for HIV-1/HBV co-infection mainly because of the HIV-1 mono-infection in a population from Burkina Faso. To our knowledge, this is the first study investigating the role of APOBEC3G variants on an HIV-1/HBV co-infection. There is a need for additional studies on a bigger population size to precise role of APOBEC3G variant in HIV-1 and HBV co-infection to comprehend the host antiviral defense system and to elaborate antiviral strategies against both HIV-1 and HBV.

## Abbreviations

APOBEC3G, Apolipoprotein B mRNA editing enzyme catalytic polypeptide-like 3G; CERBA, Pietro Annigoni Biomolecular Research Center; CI, Confidence Interval; HBsAg, Antigen anti-HBS; HBV, Hepatitis B Virus; HCC, Hepatocellular carcinoma; HCV, Hepatitis C Virus; HIV, Human Immunodeficiency Virus; HPV, Human Papilloma Virus; HWE, Hardy-Weinberg Equilibrium; LD, Linkage disequilibrium; OR, Odd ratio; SNP, Single nucleotide polymorphism; Vif, Virion infectivity factor
